# Retraction Note to: IL‑2 augments the sorafenib‑induced apoptosis in liver cancer by promoting mitochondrial fission and activating the JNK/TAZ pathway

**DOI:** 10.1186/s12935-019-1083-8

**Published:** 2019-12-30

**Authors:** Xiaoyan Ding, Wei Sun, Jinglong Chen

**Affiliations:** Cancer Center, Beijing Ditan Hospital, Capital Medical University, No 8, Jingshundong Street Chaoyang District, Beijing, 100015 China

## Retraction to: Cancer Cell Int (2018) 18:176 10.1186/s12935-018-0671-3

The editor has retracted this article [1] because Figures 2 and 4 contain duplicated and modified figures from [2–4]. The data reported in this article are therefore unreliable.

- Figure 2m in [1] contains figures that have been duplicated and modified from Fig. 2a in [2].
- Figure 2 m in [1] contains figures that have been duplicated and modified from Fig. 6j in [3].
- Figure 4g in [1] is identical to Fig. 4f in [4].
- Figure 4i in [1] contains figures that are identical to Fig. 4h in [4].

None of the authors have responded to any correspondence from the editor/publisher about this retraction.
